# X-ray Fluorescence Spectroscopy Features of Micro- and Nanoscale Copper and Nickel Particle Compositions

**DOI:** 10.3390/nano11092388

**Published:** 2021-09-14

**Authors:** Kristina A. Chebakova, Ella L. Dzidziguri, Elena N. Sidorova, Andrey A. Vasiliev, Dmitriy Yu. Ozherelkov, Ivan A. Pelevin, Alexander A. Gromov, Anton Yu. Nalivaiko

**Affiliations:** 1 Department of Functional Nanosystems and High-Temperature Materials, National University of Science and Technology MISIS, 119991 Moscow, Russia; leecrissun@gmail.com (K.A.C.); avrore@gmail.com (E.L.D.); sidelen@mail.ru (E.N.S.); raver.vasiljev@mail.ru (A.A.V.); 2 Catalysis Lab, National University of Science and Technology MISIS, 119991 Moscow, Russia; d.ozherelkov@gmail.com (D.Y.O.); i.pelevin@misis.ru (I.A.P.); a.gromov@misis.ru (A.A.G.)

**Keywords:** XRF, matrix effect, absorption, nanoparticles, Cu, Ni, powder compositions, calibration curve, calibration measurement

## Abstract

The study is devoted to X-ray fluorescence spectroscopy (XRF) features of micro- and nanosized powder mixtures of copper and nickel. XRF is a high accuracy method that allows for both qualitative and quantitative analysis. However, the XRF measurement error due to the size of the studied particles is not usually taken into account, which limits the use of the method in some cases, such as analysis of Ni-Cu mixtures and coatings. In this paper, a method for obtaining copper and nickel nanoparticles was investigated, and the XRF of powder compositions was considered in detail. The initial micro- and nanoparticles of copper and nickel were studied in detail using SEM, TEM, XRD, and EDX. Based on experimental data, calibration curves for copper-nickel powder compositions of various sizes were developed. According to the results, it was experimentally established that the calibration curves constructed for nanoscale and microscale powders differ significantly. The presented approach can be expanded for other metals and particle sizes.

## 1. Introduction

Copper (Cu) and nickel (Ni), being adjacent elements in the periodic table, both provide excellent corrosion resistance to their alloys [1,2,3]. This has contributed to their widespread use in industry, including worldwide coin production. One of the applications of both Cu and Ni is the creation of protective coatings using the cold spray deposition method [4]. This method uses powder particles as a feedstock which is deposited on the target at high speed (200–1200 m/s) and adhere to the target surface by plastic deformation of the particles [5]. Mixtures of Cu and Ni particles are used as one of the possible feedstock compositions for resisting coatings. The typical particles often used in the cold spray method are in the 5–25 µm range, but they may vary significantly depending on the purpose and equipment features [6]. The resulting coatings’ morphology, properties, and thickness vary and strongly rely on the process parameters. All this sets the problem of qualitative and quantitative control of both the initial material and deposited coatings.

Ahmed et al. [7] conducted comparative research of different quantitative chemical analyses methods of the Cu-Ni alloys, including laser-induced breakdown spectroscopy (LIBS), time of flight-mass spectrometry (TOF-MS), energy-dispersive X-ray spectroscopy (EDX), and X-ray fluorescence spectroscopy (XRF). The authors concluded that the XRF method provides accurate chemical composition detection with the lowest error of 1%. Thus, the XRF method concerning Cu-Ni is justified and effective.

XRF is often used for qualitative and quantitative elemental analysis of heterogeneous samples in many fields of science and technology [8,9,10]. This method mainly analyzes metals; hence, elements emitting X-rays below 1 keV (B, C, N, O) cannot be analyzed by XRF. It is a non-destructive, rapid, and economical method with relatively simple sample preparation requirements, suitable for various types of samples, including powdered ones. Compared to competing methods, such as atomic absorption and atomic emission spectroscopy, it has some distinctive analytical advantages: the high accuracy of measurements. Besides that, compared to the most often used methods, such as EDX and X-ray photoelectron spectroscopy (XPS), XRF allows getting information about the elemental content of the sample from the larger volume. The interaction of a material with high-energy X-ray radiation, which leads to X-ray absorption, is a base phenomenon of the XRF analytical method. The absorption/excitation effect and relaxation process lead to atoms emitting fluorescence photons with specific energy [11,12].

Quantitative XRF analysis is based on the fact that the concentration of the detected element in the sample is proportional to the intensity of the characteristic radiation emitted by this element. However, the intensity of the analytical lines is affected not only by the element’s content but also by the particles size, the chemical composition, and many experimental parameters and sample characteristics. So-called particle size effects can be sources of a significant error in the analysis of heterogeneous materials. Extreme values can even be >30% relative when analyzing samples of very different particle size distribution and light elements [13]. For many years, the X-ray fluorescence intensity from a sample increased as the particle size decreased [14,15,16,17,18,19,20,21,22]. This is due to the reduction in the size and extent of the voids in the sample. By the same reasoning, as the particle size of one of two sample components is decreased, it will yield a higher intensity relative to the component of fixed particle size. Further, if the particle size of both components is reduced, their respective intensities may increase or decrease depending on their relative absorption coefficients. It was observed that the intensities stabilize when the particle size becomes small enough.

Particle size effects are still a problem in the X-ray fluorescence analysis of heterogeneous intermediate thickness samples. Several scientific groups [14,15,16,17,18,19,20,21] have demonstrated significant variations of characteristic X-ray intensity with particle size and have developed theoretical formulae to explain them. Previous papers [17,20,21,22,23,24] described methods for particles size correction by calculations or applying correction factors. The calculation methods are based on the particle size distribution data or the ‘radiometric’ or ‘effective radiometric’ particle diameter. In other work, the particles size correction factor [25] was determined by exciting characteristic radiation of the analyte using two different radioactive sources. In other studies [26,27], a calibration method with an internal standard was proposed for pelletized samples. The authors proposed adding an internal standard using general rules, but this idea’s realization was complicated.

In [28], the influence of the micro absorption inhomogeneity of the emitter on the intensity of the fluorescence spectrum was investigated. The model considers two samples with the same composition. It was assumed that the particle size of the first sample is small and tends to zero, and the particle size of the second is larger than the thickness of the saturated layer and tends to infinity. The particles of the determined element were called “fluorescent”, and the particles of the second element were called “non-fluorescent”. Thus, if the absorption coefficient of a fluorescent element is greater than that of a non-fluorescent element, then an increase in the particle size has increased the intensity of the fluorescent element spectral line. Otherwise, the intensity of the spectrum line of the detected element increases along with the particle size increase. If the absorption coefficients of the particles are equal, then their size does not affect the intensity of the obtained spectra.

The most straightforward way to eliminate the particle size effect on the intensity of the emitted spectrum and improve the accuracy of concentration measurements is to plot the calibration curves by the standard specimens of known composition [29,30]. There are many ways to build calibration tables or curves. It is carried out using the regression set equation, the binary standard method, the semi-empirical method, the fundamental parameters method, the theoretical corrections method, etc.

In the works mentioned above [14,15,16,17,18,19,20,21,22], the particle size effect on the intensity of the emitted spectrum only at the microscale range of the particles size was studied. Specific approaches in XRF analysis may be helpful for a wide range of materials and coatings, including materials for additive manufacturing. Among them is a new field of 3D-printed multi-materials with micro and nanosized structural components. Such 3D printing methods require precise quantification of initial powders’ composition for better adjusting and controlling the printing parameters. Thus, described in the present study calibration method will define higher properties and precise dimensions of the printed details.

This paper suggests comparing the influence of the nano- and micro size powders of Cu-Ni on the fluorescence intensity by plotting the calibration curves. The method of fundamental parameters was used to plot the calibration curves. The concentration of elements in the sample was calculated by expressing the intensity of fluorescence using polychromatic primary radiation. The essential advantage of this method is that all the effects of the mutual influence of elements were considered in the analytical expression. One standard was used for calibration, along with a sample consisting only of the determining element atoms. This method takes into account the influence of the chemical composition on fluorescence intensity.

## 2. Materials and Methods

**Figure 1:**
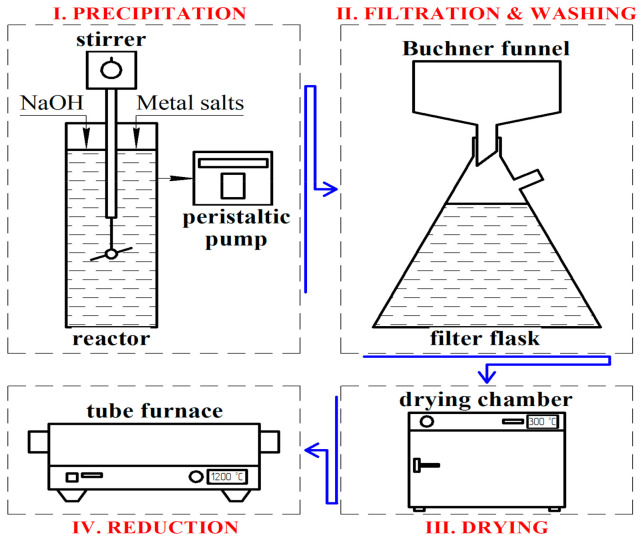
Preparation of metal nanoparticles.

Micro-sized copper powder of the PMS-1 brand (Russian State Standard # 4960-75) and micro-sized nickel powder of the PNK-1L5 brand (Russian State standard # 9722-97) were used in the study. According to the method described in the study, nickel and copper nanoparticles were obtained by precipitation of metal hydroxides (see Figure 1) from a nitrate solution [31]. A 10% solution of copper nitrate ((Cu (NO_3_)_2_ 3H_2_O) and a 10% solution of sodium hydroxide NaOH were used during the deposition of copper. Solutions were mixed under constant stirring. The mixing speed of initial solutions was adjusted to maintain the solution pH = 11. Then, the resulting hydroxide was repeatedly washed to a neutral medium and filtered off. The hydroxide was dried in a drying oven at 80 °C and reduced in a tube furnace. The subsequent reduction was carried out in a tube furnace under a hydrogen atmosphere, and passivation was carried out in a nitrogen atmosphere. The reduction temperature was 160 °C for copper and 300 °C for nickel, and the reduction time was 1 h. Then the metal powders were mixed in a powder mixer and pressed on a hydraulic press at 15 MPa.

The objects of the study were copper-nickel powders of various sizes with the following nickel content, wt.%: 0, 10, 20, 30, 40, 50, 60, 70, 80, 90, 100. The powders were weighted using a AND GR-202 analytical balance with a 0.1 mg measurement error.

The particle size was determined from micrographs using a Vega 3SB scanning electron microscope (SEM, Tescan, Fuveau, France) and a JEM-2100 transmission electron microscope (TEM, JEOL, Tokyo, Japan). At least 500 particles were measured for the analysis.

The phase analysis of the particles was carried out on a Difrey-401 X-ray diffractometer (XRD, Scientific Instruments, Saint Petersburg, Russia) with Bragg–Brentano focusing, using Cr-Kα radiations (wavelength 0.22909 nm). Before XRD analysis, the powder was pressed into a cuvette until a flat horizontal surface was obtained. The phase composition was also controlled by electronograms obtained using the JEM-2100 transmission electron microscope. The diameters of the rings were measured from the electron diffraction pattern, and the interplane distances were calculated using Equation (1):

d = C/r(1)

where C—constant determined by the microscope, r—radius of the diffraction rings, μm.

Energy-dispersive X-ray spectroscopy was used as an independent analysis and was carried out using the Advanced AZtecEnergy energy-dispersive X-ray spectroscopy (EDX, Oxford Instruments, High Wycombe, UK) attachment to the TescanVega 3SB scanning electron microscope. The acceleration voltage at the EDX analysis was 20 kV. Standard samples provided by Oxford Instruments were used to calibrate the device. Quantitative EDX analysis was performed under standard conditions based on the differences in the radiation intensity of the strongest K- or L-series line of the characteristic radiation of the element.

X-ray fluorescence analysis (XRF) was performed on a RAM-30µ analytical microprobe microscope (XRF, Scientific Instruments, Saint Petersburg, Russia) at a voltage of 30 kV and a current of 1000 µA. The energy resolution is 130 eV for Mn-Kα line. The source of the X-ray radiation was an X-ray tube with a molybdenum anode. Accordingly, the energy of the Kα-series X-ray radiation (wavelength: 0.07107 nm) was 2.8 × 10^−15^ J or 17.5 keV. The primary radiation was polychromatic, so counting the radiation energy for Kβ, Lα, and the rest of the radiation series, the energy range was 0.194–19.962 keV. Air is pumped out of the measuring chamber using a fore vacuum pump, making it possible to detect light elements, starting with sodium. The error in elements concentration depends on the accuracy of the spectral line approximation, which was done in special software for the RAM-30μ spectroscope and varied in 0.02–0.08%. The powders for XRF analysis were mixed in a certain ratio in a drum mixer for 24 h. Then the samples were prepared in a press mold at a pressure of 15 MPa. The copper and nickel concentrations in the samples were calculated in the software “exact_expert” developed by the “Scientific Instruments” JSC by the fundamental parameters method.

## 3. Results and Discussion

**Figure 2:**
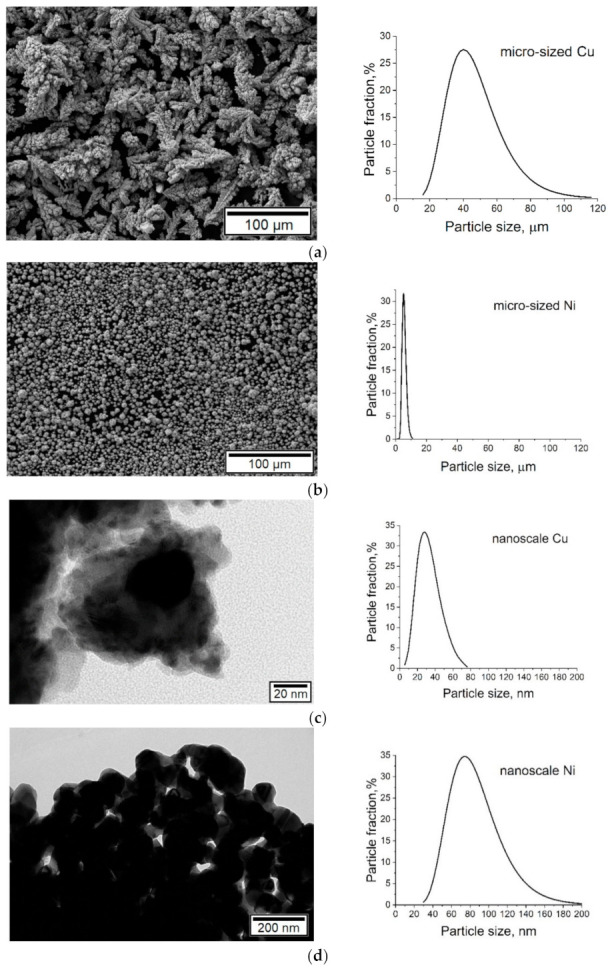
SEM images and diagrams of the particle size distribution of the initial powders: (**a**) micro-sized copper; (**b**) micro-sized nickel; (**c**) nanoscale copper; (**d**) nanoscale nickel.

SEM analysis showed that the micro-sized copper powder consisted of dendritic particles (see Figure 2a). In this sample, the length of the main axis and the span of the branches were measured. The micro-sized nickel powder (see Figure 2b), as well as nanoscale copper and nickel particles, had a rounded shape (see Figure 2c,d).

According to the particle size distribution analysis (see Figure 2), the particle size of a micro-sized copper powder varies within a fairly wide range, from 16 to 116 µm. About 70% of the crystallites varied from 26 to 66 µm with a maximum of 36–46 µm.

About 80% of the particles of the micro-sized nickel powder had a diameter from 3 to 7 µm. The distribution curve of nickel nanoparticles was quite broad, with a particle size of 29–205 nm and a maximum of 73–95 nm.

All the studied powders had a lognormal distribution and can be described by the function (2):

(2) $$f\left(D\right)=\frac{1}{Db\sqrt{2\pi }}exp\left\{-\frac{{\left(lnD-a\right)}^{2}}{2b}\right\}$$

where *a* and *b*—coefficients of the lognormal distribution, *D*—the linear size, nm.

The average linear size was calculated as the arithmetic mean of the size values for the sample (3):

(3) $$D=\frac{\sum_{i=1}^{n}D_{i}}{n}$$

The coefficients of the lognormal distribution were found (see Table 1) by analyzing the samples using the following Equations (4) and (5):

(4) $$a=\frac{\sum_{i=1}^{n}\ln(D_{i})}{n}$$

(5) $$b=\sqrt[{2}]{\frac{\sum_{i=1}^{n}{\left(\ln\left(D_{i}\right)-a\right)}^{2}}{n}}$$

**Figure 3:**
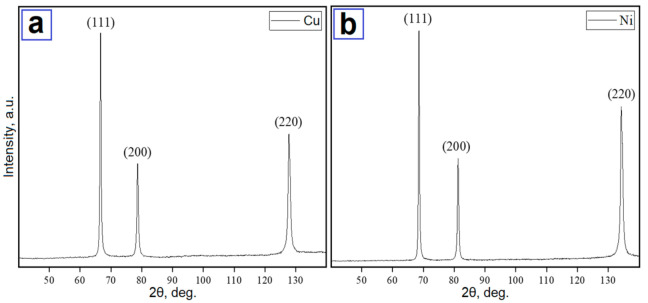
XRD patterns of nanoscale copper (**a**) and nickel (**b**) particles.

**Figure 4:**
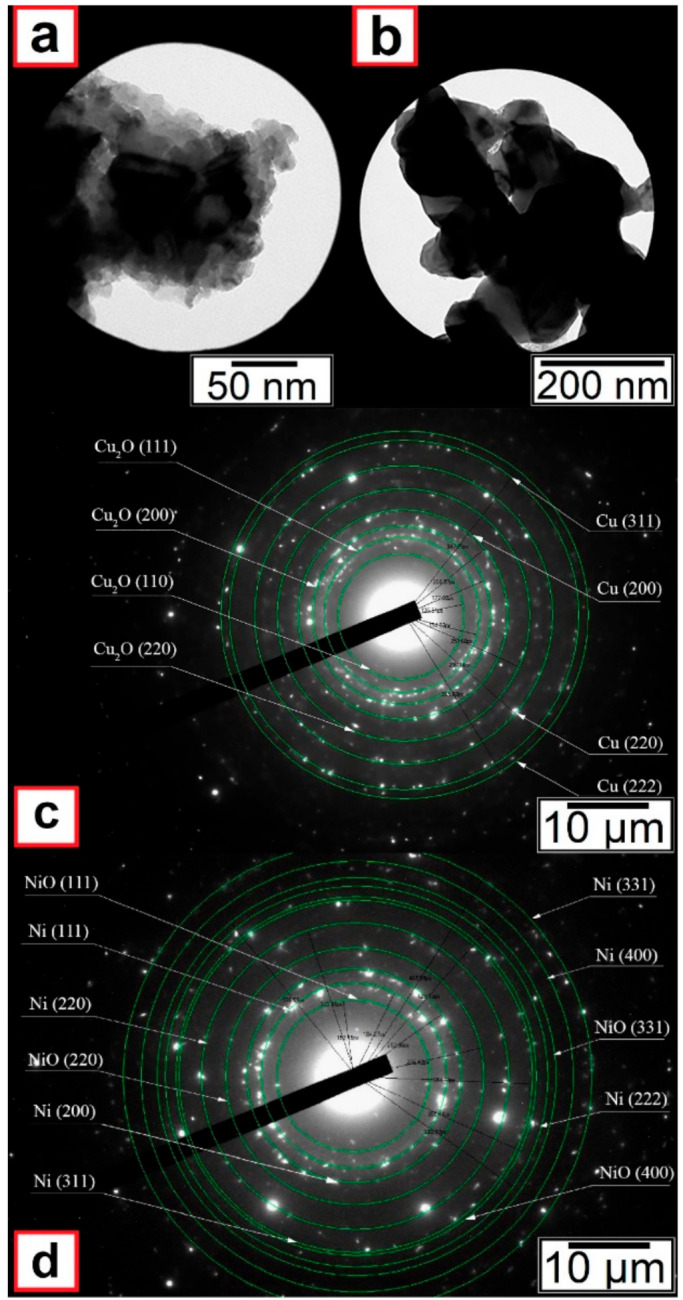
Electronograms of nanoscale copper (**a**,**c**) and nickel (**b**,**d**) particles.

Calculated particle sizes for every specified powder are presented in Table 2. Only the pure metal phase was identified on the XRD patterns of nanoscale copper (see Figure 3a) and nickel (see Figure 3b) particles. However, electron diffraction results indicated that oxides were also present in the nanoscale powders (see Figure 4).

**Figure 5:**
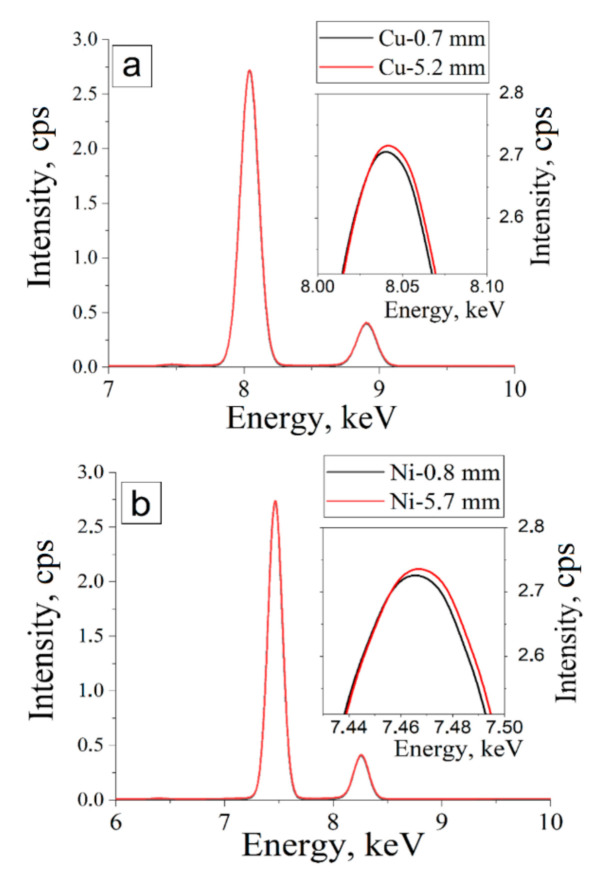
XRF spectra (Intensity, counts per second (cps) vs. Energy, keV) of copper (**a**) and nickel (**b**) particles with two different thicknesses each.

Before the XRF analysis, the background spectrum was recorded to exclude the intensities of the elements fixed by the detector from the focusing system of the device. The intensities of such background elements (Mo, Nb, Ni, Fe, Co, Cu, Zn, and Ar) were not considered during the calculations. The thickness of the saturated layer (the absorber layer thickness) required for the XRF analysis was established. For this purpose, XRF spectra were obtained from the samples with two different thicknesses. The thickness was 0.7 and 5.2 mm for nanoscale copper powder and 0.8 and 5.7 mm for nanoscale nickel powder. The obtained XRF spectra for «thin» (0.7 mm for copper and 0.8 mm for nickel) and «thick» (5.2 for copper and 5.7 for nickel) samples were superimposed on each other (see Figure 5). Captures in Figure 5 show the magnifications of the peaks and the difference between them. The obtained results showed that the 0.7 mm thickness of the absorber is sufficient for XRF analysis.

**Figure 6:**
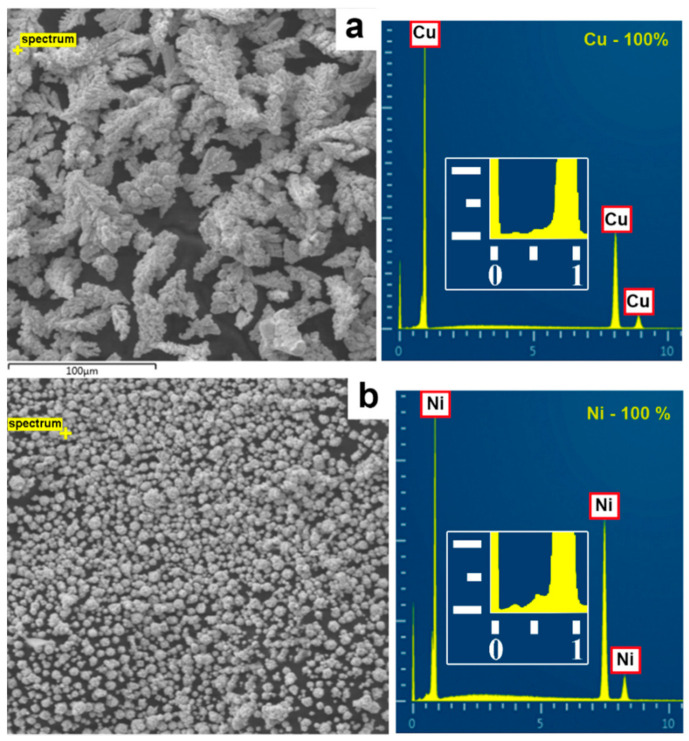

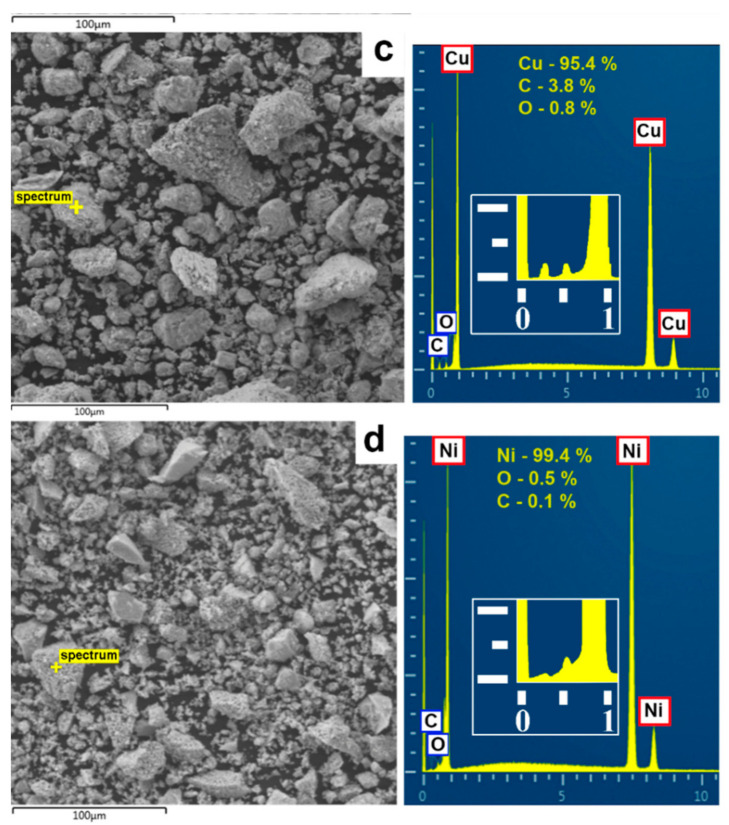
EDX analysis: (**a**) micro-sized copper; (**b**) micro-sized nickel, (**c**) nanoscale copper; (**d**) nanoscale nickel.

According to the energy-dispersive X-ray spectroscopy (see Figure 6), the content of copper and nickel in the initial micron powders was close to 100 wt.%. The analysis of nanoparticles showed the presence of oxygen: 0.8–3.9% in copper and 0.2–2.2% in nickel, which was associated with the tendency of nanoparticles to rapid oxidation.

**Figure 7:**
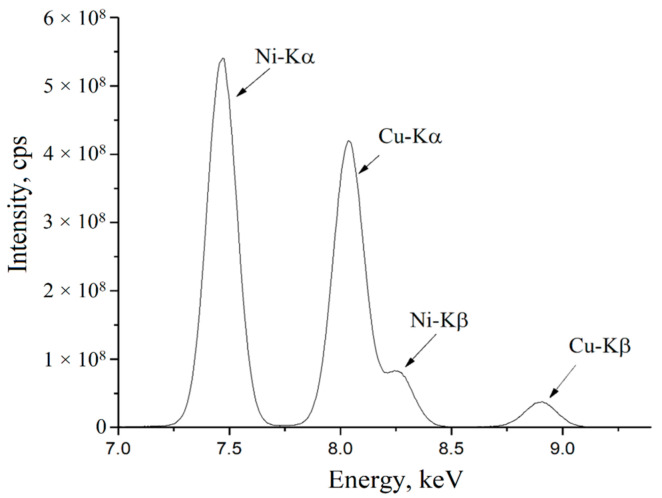
XRF spectrum (Intensity, counts per second (cps) vs. Energy, keV) of 40% Ni—60% Cu nanosized composition.

The next stage of the experiment was preparing micro- and nanoscale compositions of copper and nickel particles and their study using an analytical microprobe microscope. The obtained XRF spectrum of the nanosized composition is presented in Figure 7.

**Figure 8:**
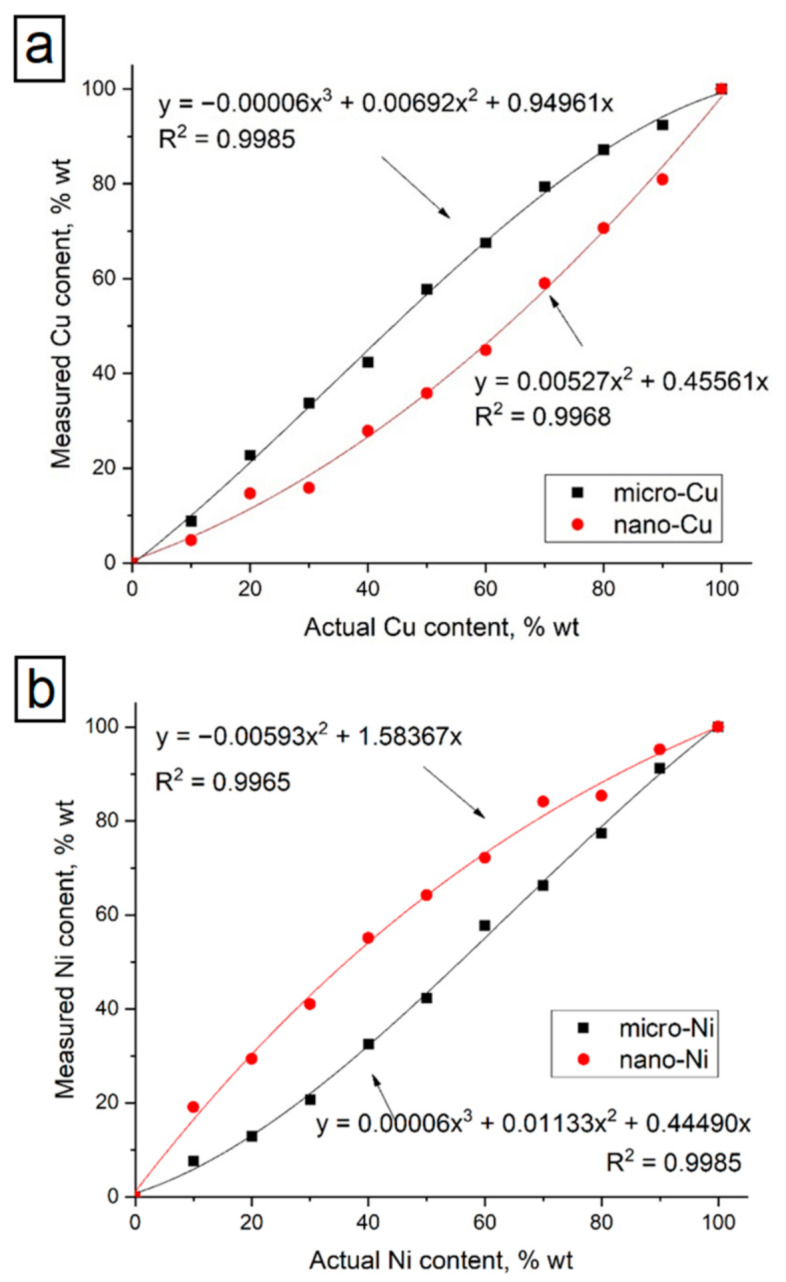
Calibration curves of micro- and nanoscale powders for Cu (**a**) and Ni (**b**).

The actual and measured content of copper and nickel in the studied samples is presented in Table 3. Calibration curves were constructed based on the obtained data (see Figure 8). A comparison of graphs for micro-and nanoscale compositions shows that the calibration curves for the considered dispersed systems differ significantly. As can be seen from the presented data (see Table 3 and Figure 8), the calibration curves for micro- and nanoscale powders demonstrated an opposite character. In the nanoscale samples, the measured copper content was lower than the actual content.

**Figure 9:**
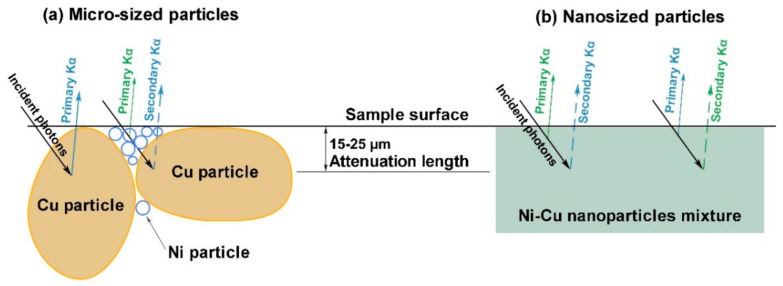
Surface morphology scheme of the Ni-Cu mixtures: (**a**) micro-sized and (**b**) nanosized powders.

Several effects may cause such behavior. First, XRF analysis is characterized by a finite penetration depth of about 100 µm for 17.5 keV incident photons. At the same time, the attenuation length is within 15–25 µm range for 7–8 keV fluorescence photons of Ni and Cu, i.e., the XRF allows mainly surface analysis in this case. The particle size distribution of the initial powders in Figure 2 demonstrates that the average size of Cu particles in the micro-sized case is far larger than Ni. On the contrary, in the nanosized case, Cu particles size is almost three times less than Ni one. Mixing, formation, and pressing procedures lead to redistribution of the particles with different sizes and specific morphology formation (see Figure 9). The difference in attenuation lengths of Ni and Cu fluorescence photons and the described morphology may affect the volume ratio of Ni/Cu and shift measured concentrations. Cu micro-sized particles allow emitting primary Kα photons from the surface (solid arrow on Figure 9) and secondary Kα photons from deeper particle areas covered by small Ni particles. Meanwhile, small Ni particles have only emitted primary Kα photons (green arrow on Figure 9a). Such surface and volumetric irregularities can explain the behavior of the corresponding calibration curves. Particle sizes are much less than attenuation length in nanosized samples, and the particle distribution is more homogeneous. The observed behavior of the calibration curves for nanosized mixtures and inversion of the micro-sized one may occur because of the described above causes.

The pressed sample’s structure may be considered Ni particles in Cu matrix or Cu particles in Ni matrix depending on the mixture concentrations, thereby matrix effect is expected. The matrix effects are absorption and enhancement of other elements with far and near absorption edges on the analyte intensity. Such effect was described and investigated in several works [32,33,34,35,36,37,38]. In absorption factor, the matrix absorbs both primary X-rays and secondary analyte X-rays if the matrix may have a smaller absorption coefficient for the analyte line radiation. In the enhancement factor, the matrix elements emit their own characteristic X-rays. The magnitude of the matrix effect varies by elements ratio in the mixture, resulting in the non-linear dependence of actual content that is clearly seen in Figure 8. Similar results for metallic powder mixtures containing Fe and Pb were observed by the authors [34,38]. It was noted that characteristic X-ray intensities of an analyte element in a composite sample might be positively or negatively affected by the chemical composition of the matrix.

Besides that, the nanosized powders are highly chemically reactive and easily absorb oxygen and carbon. The higher reactivity of Cu nanosized particles than Ni also arises due to less average copper particle size. Secondary enhancement and fluorescence scattering on the surrounding oxides and carbon detected in nanosized powder mixtures can complicate quantification. As mentioned by the authors [33], the samples’ local density and the local densities of the elements that are not detected by the XRF method play an important role in the scattering and secondary enhancement, thus affecting the quantification of the main elements. A similar impact of oxides on the analytical results was described by the authors [39]. The precision of XRF analysis was improved to 1% by fusing the powder samples compared with that of 7% for initial powders. Such improvement was associated with less non-uniformity, homogeneous distribution of the elements, and consequently lower matrix effect.

It is still a big challenge to improve the accuracy of the analysis by including the knowledge on secondary enhancement and scattering and adapt it to the specific samples. Some theoretical [32,37] and experimental [34,35] models for correcting secondary enhancement mechanisms on the total fluorescence intensity and matrix effect were investigated. Theoretical methods are based on the influence coefficients and fundamental parameters. In contrast, experimental methods are based on intensity ratio and can correct both matrix and physical effects (particle size, surface effects, etc.) However, these correction methods are very much dependent on the nature of the specimens and their composition ranges. Authors [40] conclude that using reference calibration samples with similar morphology is necessary to obtain accurate XRF results for such problematic specimens as powders.

All mentioned effects that give total error in measured content of the elements can be successfully accounted for by calibration curves construction using the described methodology. It should be noted that conventional tabulated values of the mass absorption coefficients were obtained based on coarse-crystalline samples calculations. In the case of nanoscale materials, these coefficients will differ. Such simplification introducing an error to practical analytical situations and cannot account for all variations of materials conditions. The authors [32] noted that influence coefficients used during calibration are usually calculated for a given composition range from physical values like absorption coefficients and fluorescence yields. Nowadays, software and XRF spectrometers use calibrations based on homogenous specimens with flat and polished surface and an infinite thickness. The accuracy during practice analysis depends on the nature of the specimens being analyzed. These influence coefficients are not constant and dependent on the concentration ranges, samples density, and matrix effects.

In general, the obtained data can be predicted by analytical calculations; thus, the discrepancy between the actual and measured content was experimentally confirmed in this study. As noted above, the most significant discrepancy in the measurements was found in the case of nanoparticles analysis. Based on this, it was recommended to carry out a similar procedure with the calibration curves in the study of nanomaterials by the XRF method. Such a preparatory stage will allow for higher measurement accuracy.

These research results can be useful in metallurgy and geology to improve the accuracy of the XRF analysis. Since, at the moment the calibration curves are constructed only for powders of two metals, it is also necessary to see how the results change in a wide range of particle sizes and other elements. In general, the work has prospects for further development and practical application in other metals and different particle sizes. Besides that, this research shows the necessity of developing new software packages based on the new approaches corresponding to modern analytical needs.

## 4. Conclusions

This paper presents a study of micro- and nanoscale compositions consisting of copper and nickel particles. The initial powders’ particle size distribution, chemical, and phase composition were studied in detail using SEM, TEM, XRD, and EDX. Studies of the elemental composition of micro-and nanoscale copper-nickel compositions using XRF analysis have been carried out. According to the obtained data, calibration curves for the measured and actual content of nickel and copper were constructed. The obtained calibration curves indicated that the XRF analysis data for micro- and nanoscale samples differ significantly. The observed differences are due to particle size factor, different attenuation lengths of Ni and Cu, matrix effect, high reactivity, and tendency to oxidation of nanoparticles. Each factor can play a pronounced role, and their combination explains the observed behavior of calibration curves. The presented experimental method allows to consider all factors and increase analysis accuracy. Calibration curves can be used to characterize copper and nickel powders using XRF data, and the presented approach can be expanded for other metals and their mixtures with different particle sizes.

## Figures and Tables

**Table 1:** Coefficients of the lognormal distribution.

Sample	Coefficient	Measured Value	Approximation Coefficient (R^2^)
Micro-sized copper (see Figure 2a)	*a*	3.807	0.988
	*b*	0.341	
Micro-sized nickel (see Figure 2b)	*a*	1.692	0.991
	*b*	0.238	
Nanoscale nickel (see Figure 2c)	*a*	4.412	0.971
	*b*	0.323	
Nanoscale copper (see Figure 2d)	*a*	4.037	0.967
	*b*	0.378	

**Table 2:** Dimensional characteristics of the initial powders.

Powder	Measurement Parameter	Minimum	Maximum	Average
Micro-sized	Length of Cu particles, µm	16.01	109.8	47.8
	Span of Cu particles branches, µm	9.6	62.7	23.2
	Diameter of Ni particles, µm	1.7	14.9	5.6
Nanoscale	Diameter of Cu particles, nm	6.0	77.0	24.0
	Diameter of Ni particles, nm	30.0	202.0	87.0

**Table 3:** Calibration table of Cu/Ni composition measured by XRF: actual content vs. measured content.

Micro-Sized Compositions				Nanoscale Compositions			
Actual Content		Measured Content		Actual Content		Measured Content	
Cu, %	Ni, %	Cu, %	Ni, %	Cu, %	Ni, %	Cu, %	Ni, %
100	0	100.00	0	100	0	100.00	0
90	10	92.46	7.54	90	10	80.95	19.04
80	20	87.22	12.78	80	20	70.66	29.33
70	30	79.40	20.60	70	30	58.98	41.04
60	40	67.52	32.48	60	40	44.90	55.09
50	50	57.72	42.28	50	50	35.80	64.20
40	60	42.32	57.68	40	60	27.87	72.12
30	70	33.74	66.26	30	70	15.88	84.11
20	80	22.70	77.30	20	80	14.65	85.34
10	90	8.84	91.16	10	90	4.78	95.21
0	100	0	100.00	0	100	0	100

## Data Availability

The data presented in this study are available on request from the corresponding author.
